# Mechanical characterization & regression analysis of *Calamus rotang* based hybrid natural fibre composite with findings reported on retrieval bending strength

**DOI:** 10.1038/s41598-024-53570-7

**Published:** 2024-02-16

**Authors:** K. S. Lokesh, D. Shrinivasa Mayya, H. L. Yashwanth, I. S. Sharanya, Hrithika Nikam, K. L. Channa Keshava Reddy, Shashank Kumar

**Affiliations:** 1 Aeronautical Engineering, Srinivas Institute of Technology, Mangalore, 574143 India; 2 Mechanical Engineering, RNS Institute of Technology, Bangalore, 560098 India

**Keywords:** Biomaterials, Structural materials, Mechanical engineering

## Abstract

Research on Bio-based natural fiber material promoted the development of reinforcement and expand their possible structural applications. In this study, fibers are extracted from the stem of *Calamus rotang* (common rattan-Indian Species). Further, the fiber is processed to get novel hybrid combinations with glass fibers by manual hand lay-up technique. Three sets of samples were prepared for the different volume fractions of 60:40, 30:30:30, and 60:32:8 of glass fiber/epoxy as neat composite sample (NCS), a hybrid combination of *C. rotang* /glass fiber with epoxy as modified reinforced composite sample (MRCS) and glass fiber/epoxy with calamus stem powder as modified matrix composite sample (MMCS) respectively. Mechanical tests including tensile, flexural, impact, and ILSS tests are conducted as per ASTM Standards. Comparative studies have been done to evaluate the effect of novel species of *C. rotang* on mechanical properties with neat GFRP composites. Addition to this regression analysis has been carried out to achieve the experimental correlation for tensile and bending tests. Microstructural analysis for all the tested samples has been done to assess the fracture mode. Novel findings on retrieval bending strength for MMCS has been reported for the first time for composite materials. Study proves that novel species have a significant impact on the basic properties of materials.

## Introduction

Utilizing the most commonly available natural fibres which are preferred in developing lightweight structures reduces the dependency of composite manufacturing on synthetic and expensive fibre. Non-structural applications especially in automotive industry with the aspect of achieving light weight natural fibres are forefront to represent the available choices^1,2^. Extensive usage is mainly due to high performance of these materials compare to natural fibers and also random fibres with fibre mats like chopped strands are preferrable choice due to the fact that availability, cost effectiveness though they do not have specific directional stress distribution^3^. In the available class of materials green materials such as bamboo and cane wood are interesting species contributed better mechanical properties. it is observed from the results that bending strength is improved and bit more as compared with bamboo fibers^4^. Additionally, use of abundantly available agriculture-based materials such as jute, silk waste^5^, banana fibre^6^ based composites deliver the enhanced mechanical performance. Utilization of fish scale and cocopeat^7^ witness the possible transformation of waste in to light weight structures. Using rattan cane with fiber fraction range from 5 to 30% with epoxy matrix were significantly contributes to abrasion, water absorption as compare to neat epoxy samples, within the reinforcing range of 10% of rattan fibres yield better abrasion resistance compare to the rest^8^. The chemical composition of similar species reports cellulose, hemicellulose, lignin percentage of 42%, 20% and 27% respectively from the previous studies where fiber extraction from manau rattan species were developed and examined for thermal and mechanical testing where these species attributed for better thermal resistance up to 300^0^c and enhanced mechanical properties^9^. Impact of fibre length, shape, proportion and interfacial adhesion also directs composite performance^10^. Effect of corn cob particles on mechanical behaviour of e-glass fibre epoxy hybrid composites were studied^11^ where the composition of 25:5:70 yield better impact resistance where 27.5:2.5:70 yield better tensile modulus and tensile strength confining the range of corn cob inclusion is optimal for 2.5–5% to obtain better results. Coconut coir-based composites were prepared in different weight percentage to evaluate the basic mechanical properties. The weight proportion considered was 0%, 2.5%, 5%, 7.5% with carbon fibres. Out of all the combinations 10% by weight yield the optimal test results in terms of impact and creep resistance^12^. Addition to these categories, ramie, basalt fibre reinforced with polyester composites enhance greatly on mechanical properties. optimal piling sequence of the sample contribute better tensile, flexural and hardness of the samples^13^. With probable dominance of natural fibres not just in automotive sectors but also for other industry explorations^14^. There should be a need of exploring natural fibres which renders their response more significantly. The reported literature suggests that no potential work has been carried out relating to the extraction of the novel species of rotang calamus fibers as reinforcement with glass fibers and optimal percentage of fillers with epoxy to form a hybrid composite followed by characterization of the developed samples. Hence the present study aims to develop bio-based natural fiber material as reinforcement via threaded and powder forms through manual hand layup route and the study aiming to achieve over utilization of synthetic fibres and polymers and also to assess the mechanical properties of developed composites viz., tensile, flexural, Impact, Interlaminar shear strength properties followed by characterization studies.

## Materials used and methodology

- Epoxy (LY556)/Hardner (TETA)
- *Calamus rotang*
- E-glass fiber(CSM)

### Sample preparation steps

**Figure 1:**
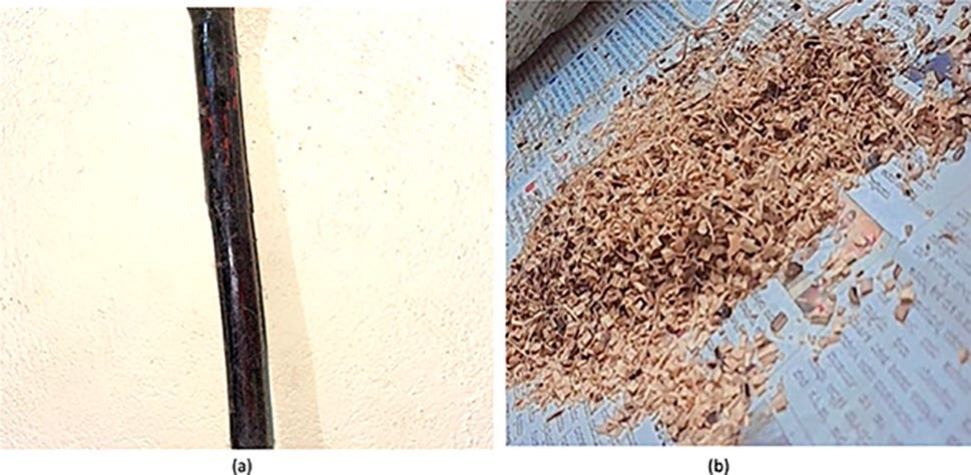
(**a**) Stem of *Calamus rotang* and (**b**) Powdered Stem of *Calamus rotang.*

**Figure 2:**
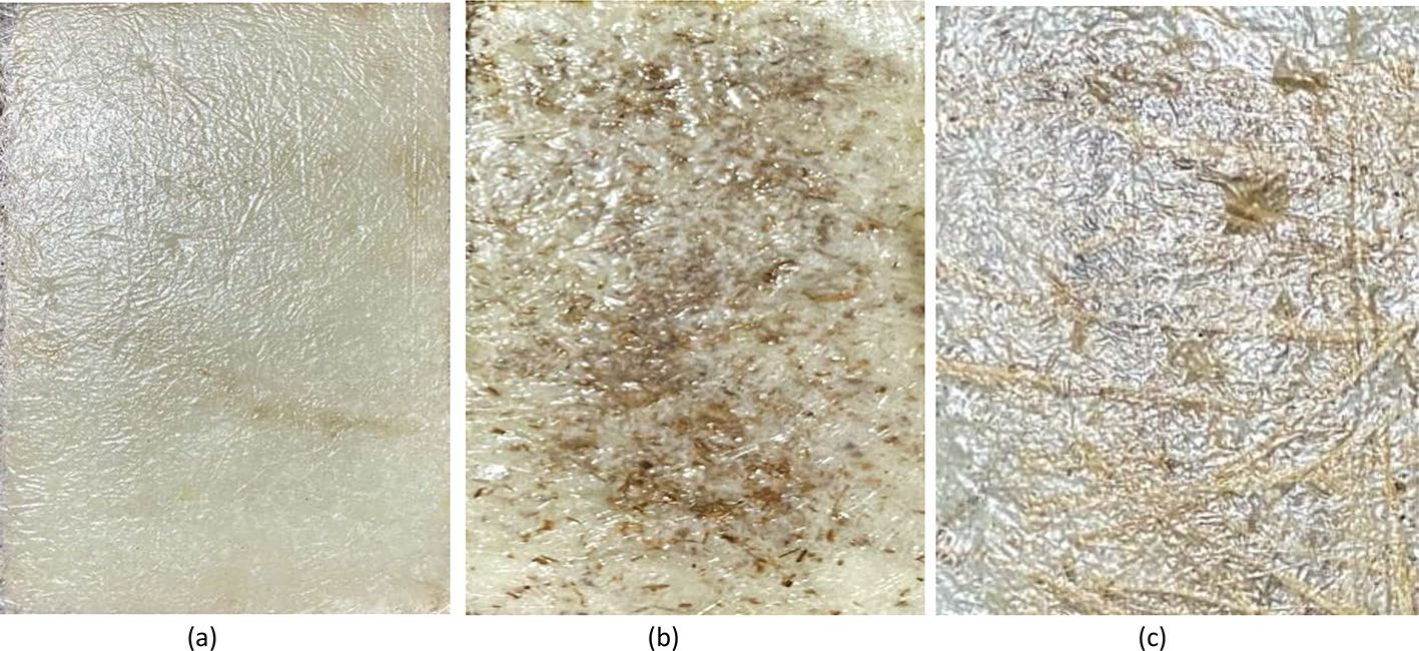
(**a**) Neat Composite Sample (NCS), (**b**) Modified Matrix Composite Sample (MMCS) & (**c**) Modified Reinforced Composite Sample (MRCS).

**Figure 3:**
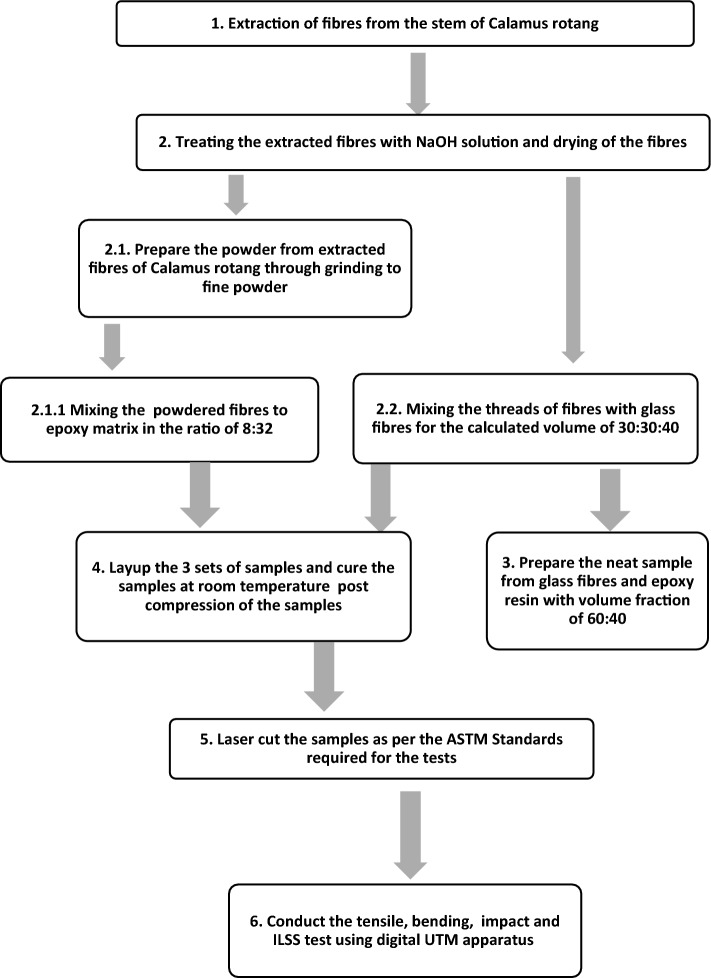
Work flow for sample preparation.

Extraction of fiber; *C. rotang* stem as shown in Fig. 1a is collected from the local market in Chamarajanagar, Mysore, Karnataka, India. Though the calamus species is majorly found in Mahadeshwara and gundya forest, Mangalore, it is commercially made available for the usage (where this is locally termed as nagarabetta in native language which is normally used for pooja purpose). Impurities and contaminants are removed by cleaning the fiber in water. Fiber is naturally extracted in the form of thread and powder as shown in Fig. 1b. Sample preparation was done by hand layup method for preparing 3 different sets of samples indexing Neat Composite Sample (NCS), Modified Reinforced Composite Sample (MRCS) and Modified Matrix Composite Sample (MMCS). Prepared composite plates are shown in Fig. 2. Workflow for the same has been indicated with the Fig. 3.

### Test conducted

Four different tests have been conducted as per ASTM standards^15^ to determine the mechanical properties of the prepared samples.

#### Tensile test

**Figure 4:**
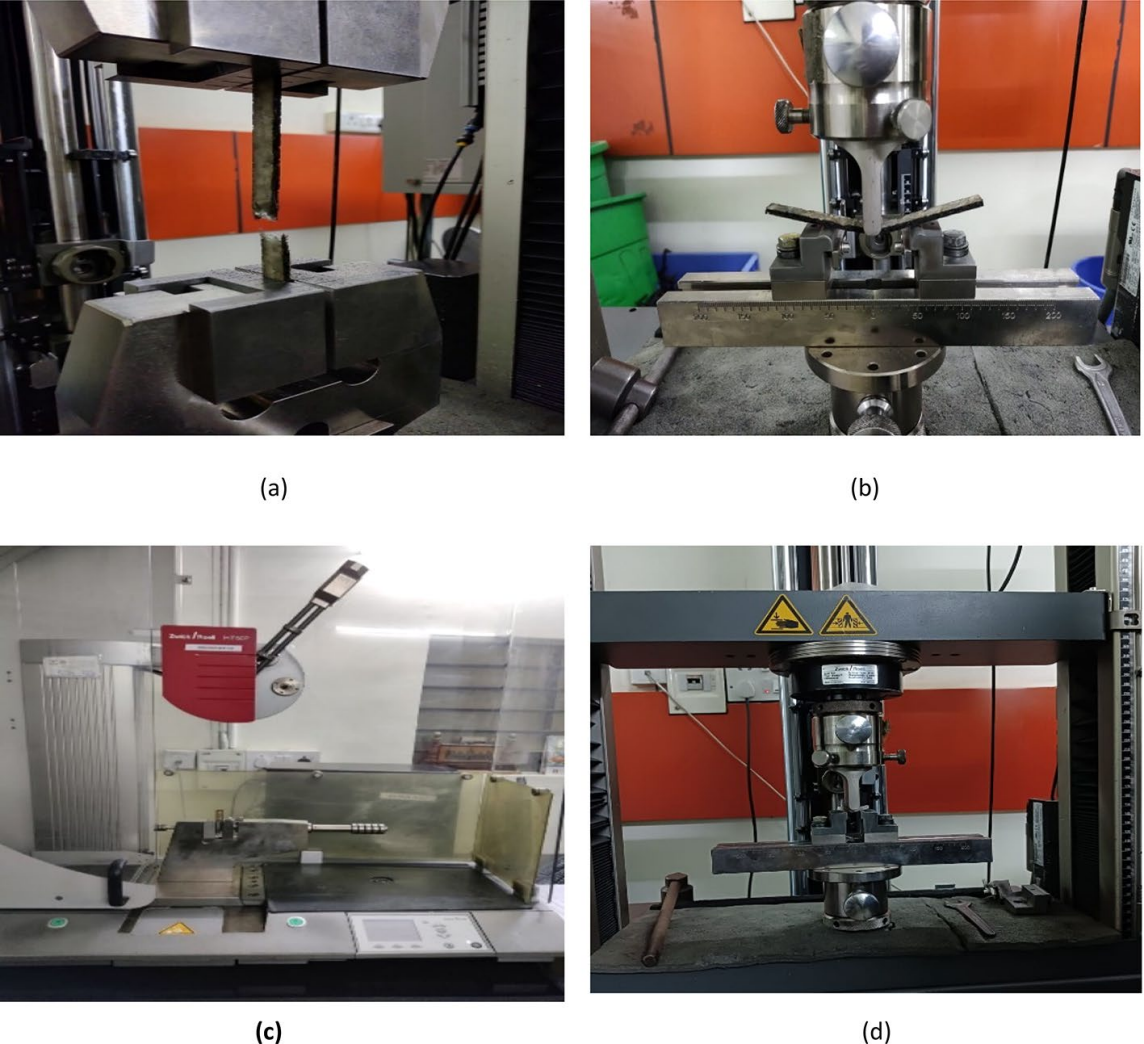
Mechanical testing of the prepared samples for (**a**) tensile (**b**) Bending, (**c**) Impact (d) ILSS.

Tensile test was conducted with the help of digital UTM device (ZWICK ROELL, Z020) with Load cell capacity of 20 KN. Specimen is mounted in the vertical direction and Maximum load taken, Modulus and Ultimate Tensile strength of the different combinations is documented by performing tensile test as shown in Fig. 4a.

#### Bending test

Three-point bending test is performed on the samples to determine the maximum load taken, flexural modulus and Flexural strength for the different combinations of samples. Bending test was conducted with the help of digital UTM device (ZWICK ROELL, Z020) with Load cell capacity of 20 KN. Specimen is placed in the test bed for bending test with testing condition of the specimen as shown in Fig. 4b

#### Impact test

To determine the amount of energy absorbed by the samples during fracture, impact test is performed as shown in Fig. 4c. Impact test was conducted with the help of digital pendulum impact device (ZWICK ROELL HIT 50P) with the nominal load capacity of 5.5 J and the impact velocity of 3.458 m/s.

#### ILSS test

To determine interlaminar shear strength for the prepared samples, ILSS test has been conducted with the help of ILSS testing device (ZWICK ROELL, Z020) with the Load cell capacity of 20 KN and applied preload of 2N. Testing with fixed speed rate 1mm/min was set while testing till the failure of the specimen was achieved. Loading condition for the tested samples as shown in Fig. 4d.

### Declarations

The present study is complied with relevant institutional, national, and international guidelines and legislation.

### Required permissions

Relevant permits/permissions/licences were obtained.

### Ethical approval

This article does not contain any studies with human participants.

### Human and animal rights

This article does not contain any studies with human or animal subjects performed by any of the authors.

## Results and discussion

### Tensile testing

Experimental study aims to investigate the effect of *C. rotang* based natural fibers and powders on reinforcements as well as matrix of glass fabric reinforced plastics. Tensile, flexural and impact tests have been done as per the ASTM standards to evaluate the basic mechanical properties of the said combination. Three different test samples were considered for testing such as Chopped glass fiber/epoxy combination as pure sample, modified matrix laminates (MMCS) and modified reinforcement laminate (MRCS) samples are tested for basic mechanical tests and consolidated test results has been discussed in detail and also comparative study has been made in to understand the better combination of the tested samples.

**Figure 5:**
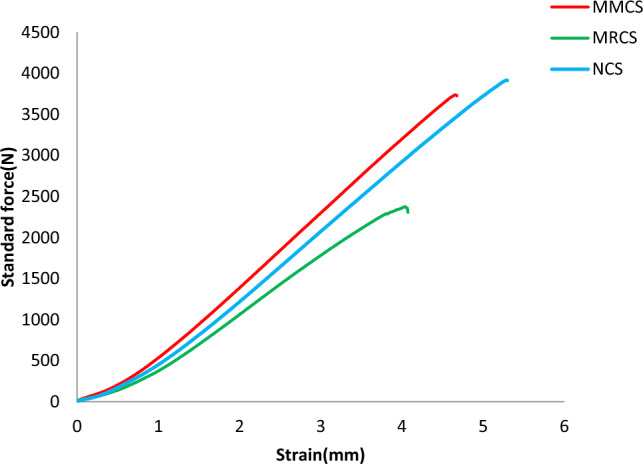
Load (N) v/s Deformation (mm) of tensile testing.

Figure 5 depicts the load and deformation for 3 different sets of tensile tested specimens. Results revealed that significant impact of *C. rotang* stem extracts through modified matrix system in enhancing the axial load taking capabilities of the sample. Table 1 depicts the obtained tensile test results. For tensile loading, modified matrix combination records nearly 36.4% increase in load bearing ability of the loaded micro fillers in to matrix system which can permit it up to 8% above which digesting the loaded fillers is difficult with reinforcements.

**Table 1:** Results obtained from tensile testing.

Sample indexing	Max load (N)	Tensile modulus (Mpa)	Ultimate tensile strength (Mpa)	Strain at break (%)
Neat sample (NCS)	3916.92 ± 78.01	596.96 ± 9.21	104.13 ± 2.06	5.3
Modified reinforcement (MRCS)	2376.47 ± 41.21	481.10 ± 8.01	55.85 ± 1.87	4.07
Modified matrix laminates (MMCS)	3737.51 ± 69.33	727.78 ± 12.09	82.85 ± 1.95	4.67

Better performance of composites under tensile loading is due to excellent interfacial adhesion between the constituents leads to lower void content^16–18^. However, the neat glass fabric sample reports the peak load value, upon modifying the matrix the sample records higher stiffness value of nearly 22% as compare to the neat and 51.2% better as compare to modified reinforcements.

**Figure 6:**
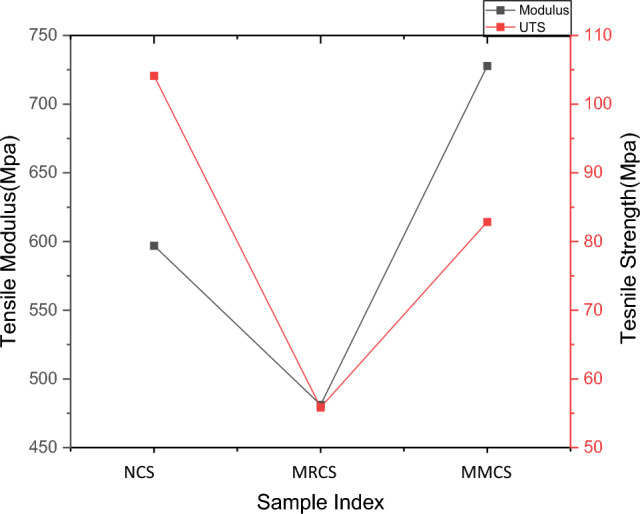
Cmparison of tensile Characteristics.

Compare to ultimate tensile strength it is found interesting that modified matrix sample performs nearly 49% greater value as compare to that of reinforcements, virgin sample remain 25% ahead of matrix modified sample. It’s clear that modified reinforcements of *C. rotang* extracts remain at the last in performing against tensile loading due to the fact that random discontinuous strands do not influence in enhancing interfacial bonding with the matrix due to imperfect stacking and these findings are in consistent with the previous study claims the poor performance of twisted non-aligned discontinuous fibres delivers poor performance due to weaker fibre-resin bond strength was reported^19^. Comparison of tensile characteristics is reflected from Fig. 6.

Obtained strength results for the present work remain excellent as compared with the previous work done on evaluating the performance of coconut fibre/epoxy and coconut/polyester reinforced composites and also banana/jute/epoxy-based composites depicting the lower value of UTS^12,18^. Interestingly composites made of carbon/banana/jute/epoxy-based yield the UTS value of 59.58MPa which is similar for the combinations of composites considered for the present work^20^.

#### Regression analysis

**Figure 7:**
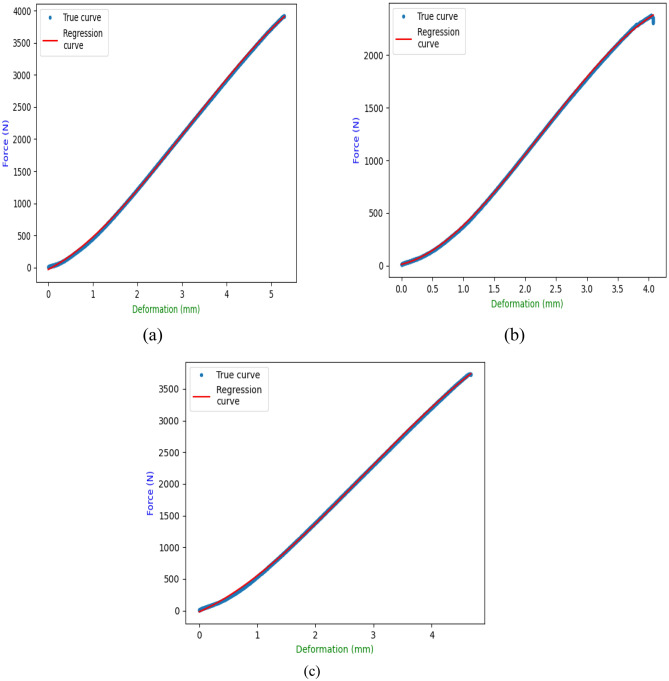
Regression results of (**a**) NCS (**b**) MRCS (**c**) MMCS.

Regression analysis of tensile testing of neat sample (NS), MRCS (10% Calamus fiber), MMCS (10% Calamus powder) has been reported in the Table 2. Figure 7a–c represents obtained plots of comparison between experimental (true) curve and regression curve. Quadratic and Cubic model results for the 3 different set of samples obtained to perform statistical analysis and to draw the correlation with experimental results. Cubic regression model reports greater correlation with experimental as compare to quadratic model. Same has been reported^7^ while assessing the fish scale and coconut shell-based bio composites. For the cubic model to be considered all the 3 set of samples shown excellent agreement of depicting 99.99% with experimental values where the slight deviation was found for quadratic model ranging from 99.50% to 99.83% for all sets of samples.

**Table 2:** Regression results for tensile testing of NS, MRCS, MMCS samples.

Specimen	Regression	Regression coefficient (R^2^)	Regression equation
NCS	Quadratic	0.9980	28.1985 X^2^ + 644.1925X + − 149.7976
	Cubic	0.9999	− 17.9652X^3^ + 169.0350X^2^ + 352.9649X + − 31.6194
MRCS	Quadratic	0.9950	30.1550X^2^ + 518.7633X + − 100.1003
	Cubic	0.9999	− 39.8464X^3^ + 271.1804X^2^ + 137.7453X + 13.0090
MMCS	Quadratic	0.9983	34.6552X^2^ + 690.5257X + − 124.0924
	Cubic	0.9999	− 22.5190X^3^ + 190.9079X^2^ + 403.6412X + − 19.6604

#### SEM analysis

**Figure 8:**
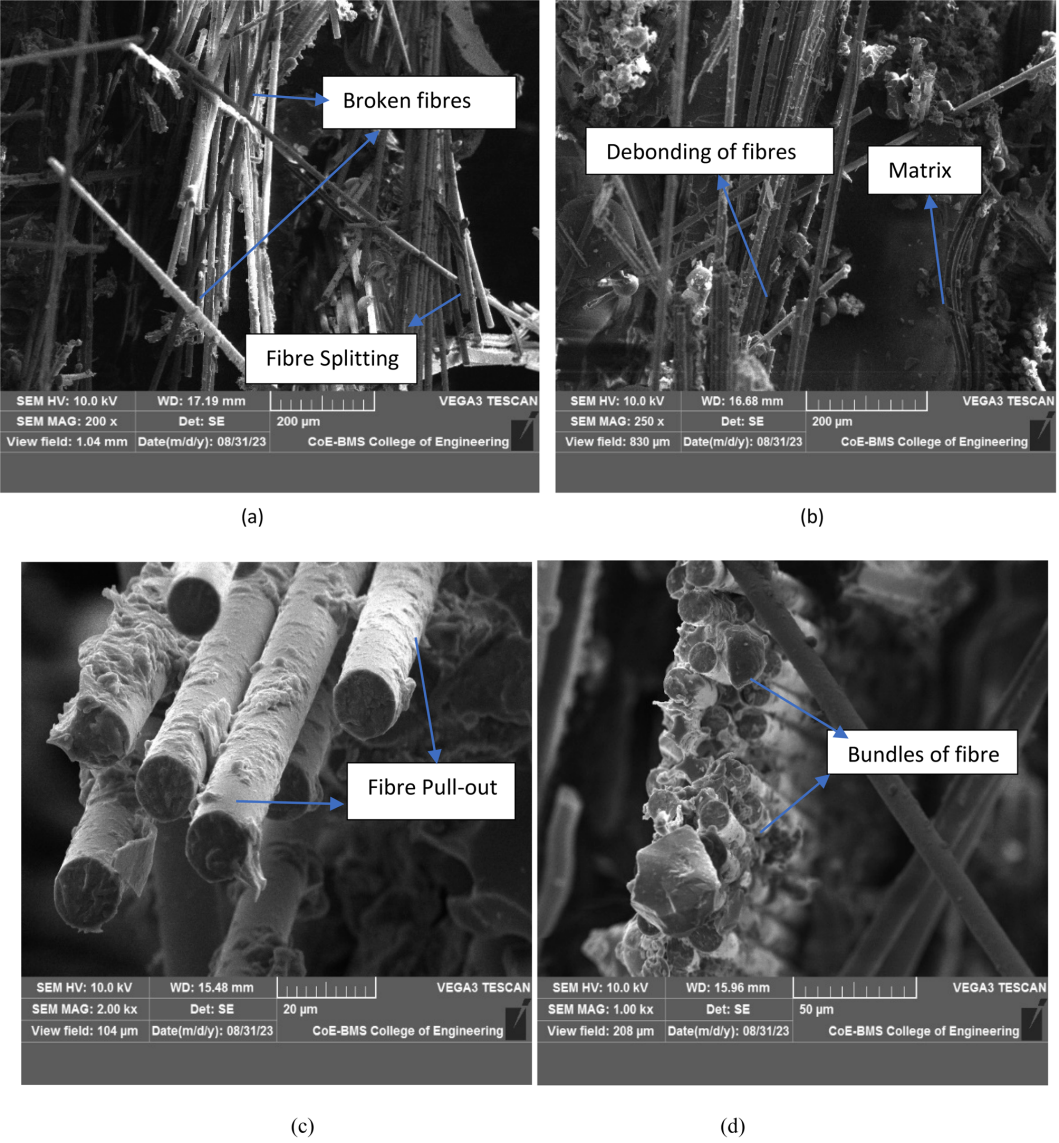
Fractured surface for (**a**) NCS & (**b**) MRCS (**c**, **d**) for MMCS.

Above micro structure resembles the fractured areas of the tested samples in which the damage is observed for the NCS and MRCS is shown in Fig. 8a,b. Splitting of fibres with changed orientations in the random manner was noticed. brittle mode of fracture was sharply highlighted with due adhesion of matrix with glass fibre and aligned calamus fibre. Fractured sample of MMCS depicting the fibre pull out as shown in Fig. 8c with respect to matrix. This also indicating the better fibre matrix interaction with fibre pull out from the bonded matrix suggesting the debonding of the fibres from the matrix at peak loads^8^ and bundled fibres for matrix modified with calamus stem powder is shown in Fig. 8d. Neatly aligned reinforcement which is found to be in good agreement with matrix and fibre combinations for MMCS reflects the better ultimate tensile strength as compare to MRCS.

### Flexural testing

**Figure 9:**
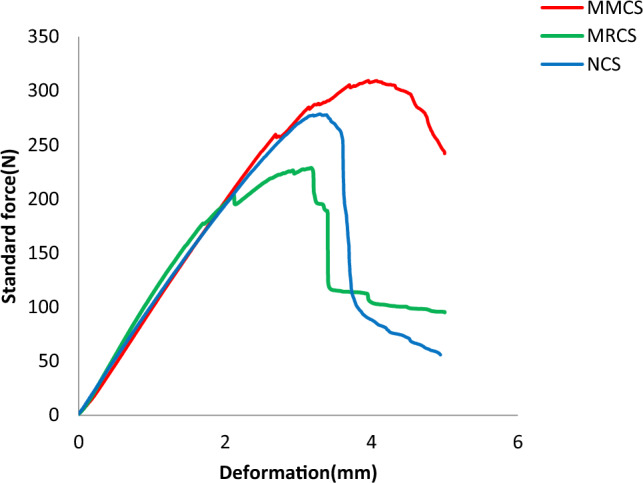
Load (N) v/s deformation (mm) obtained from flexural testing.

**Figure 10:**
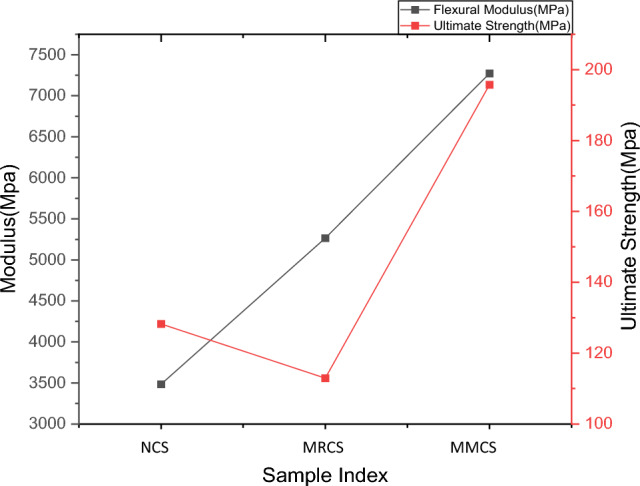
Comparison of flexural characteristics.

Load and deformation for the samples tested under bending load is as shown in Fig. 9 and Comparison of bending performance over 3 different samples is depicted from the Fig. 10.

For bending test, out of three variations, samples with modified matrix system resists more value of bending load as compare to the rest of the combinations, its observed that this proportion yield maximum bending load of 10.11% higher than that of neat sample whereas the same parameter records 35.4% higher than that of sample with modified reinforcement. Load taking capability of MMCS is due to uniform mixing of powdered *C. rotang* stem with matrix and better interfacial adhesion between the constituents. The Response of flexural stiffness for the modified reinforcement seems to better about 51% than that of matrix modified sample though the response in stiffness value report behind of 38 % with that of pure sample. Obtained bending test results are tabulated from the Table 3.

**Table 3:** Results obtained from the flexural test.

Sample indexing	Max load (N)	Flexural modulus (Mpa)	Bending strength (Mpa)
Neat sample (NCS)	278.47 ± 5.45	7270.40 ± 85.21	196.75 ± 3.21
Modified Reinforcement (MRCS)	218.76 ± 4.06	2270.26 ± 27.33	113.95 ± 2.82
Modified Matrix laminates (MMCS)	309.48 ± 6.33	3490.10 ± 39.08	128.23 ± 2.54

The similar trend was never been observed while finding its strength where the modified matrix performs better with 13.6% more strength than that of modified reinforcement sample though neat sample is 52% ahead of it. The penultimate combinations of these variations firmly give the indications to roughly choose to modify physicochemical features considered to be greater impact on bending strength of these bio composites. Influence of basalt/ramie hybrid reinforcement on bending strength of polymer composites found to be in consistent with the present results^21^. Interestingly composites-based carbon/banana/jute/epoxy reports lower value of bending strength compare to the present work which denotes the reinforcing effect of rattan fabric threads on glass fibres and influence of rattan stem powder on matrix delivers enhanced resistance to bending load.

#### Regression analysis

**Figure 11:**
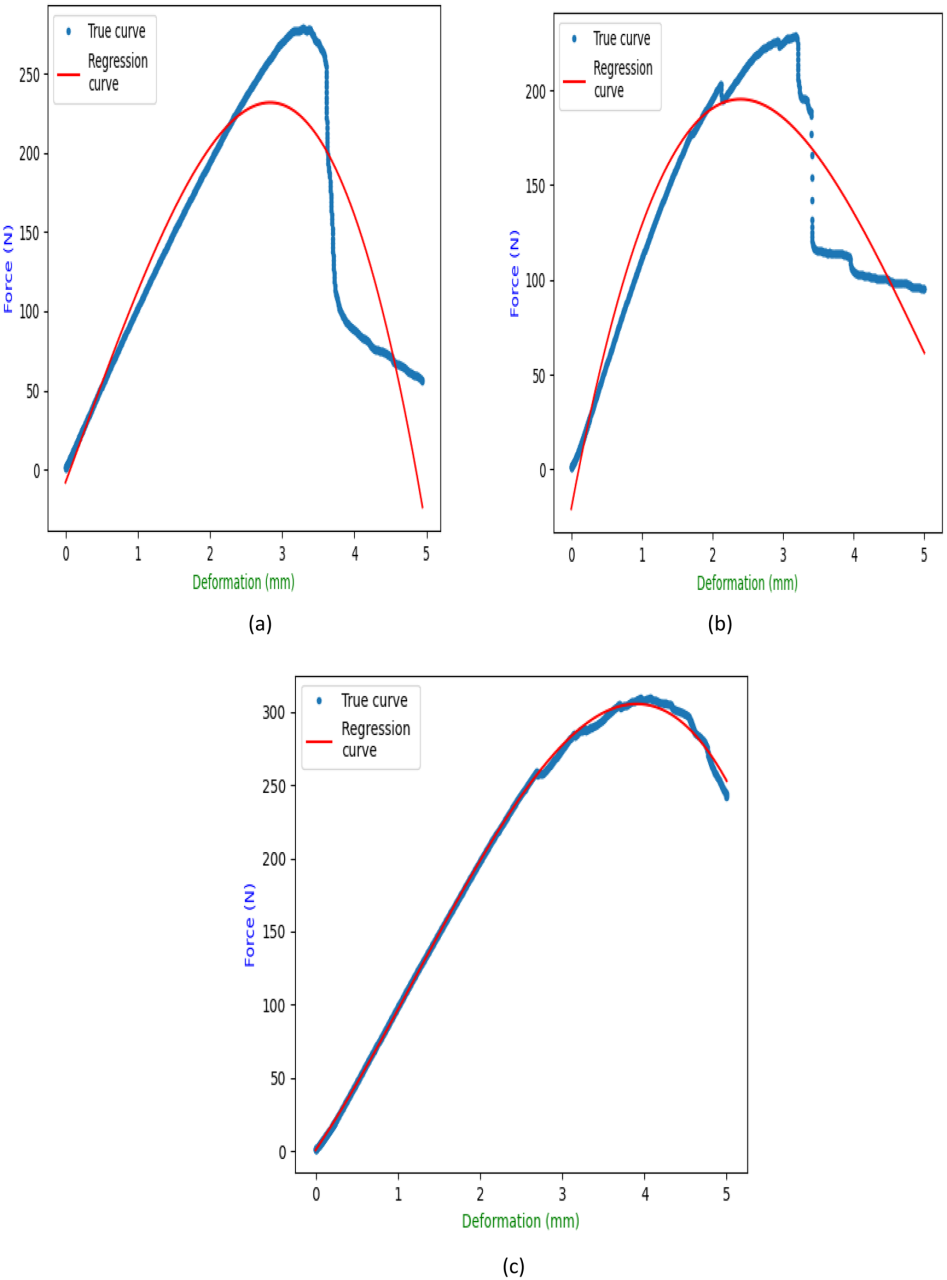
Regression results of (**a**) NCS (**b**) MRCS (**c**) MMCS.

Regression analysis of bending testing of neat sample (NS), MRCS (10% Calamus fiber), MMCS (10% Calamus powder) has been reported in the Table 4. Figure 11a–c represents obtained plots of comparison between experimental (true) curve and regression curve. Quadratic and Cubic model results for the 3 different set of samples was framed to perform statistical analysis and to draw the correlation with experimental results. Cubic regression model reports greater correlation with experimental as compare to quadratic model. Acceptable range of correlation ranging from 85.49% to 99.22% with experimental values for 3 set of samples when it is performed for cubic model where this accuracy was bit reduced for the same with the range of 83.16%–98.16% when performed for quadratic model.

**Table 4:** Regression results for flexural testing of NS, MRCS, MMCS samples.

Specimen	Regression	Regression coefficient (R^2^)	Regression equation
NS	Quadratic	0.8316	− 38.0802X^2^ + 197.1345X + − 29.7238
	Cubic	0.8549	− 5.5569X^3^ + 1.6036X^2^ + 124.6618X + − 8.0207
MRCS	Quadratic	0.8775	− 28.9075 X^2^ + 153.2204X + − 6.5233
	Cubic	0.8946	3.5792 X^3^ + − 54.7879 X^2^ + 200.9317X + − 20.8668
MMCS	Quadratic	0.9870	− 17.1790 X^2^ + 146.6393X + − 18.3611
	Cubic	0.9992	− 4.7708 X^3^ + 17.2918 X^2^ + 83.2193X + 0.7599

#### Retrieval bending strength

**Figure 12:**
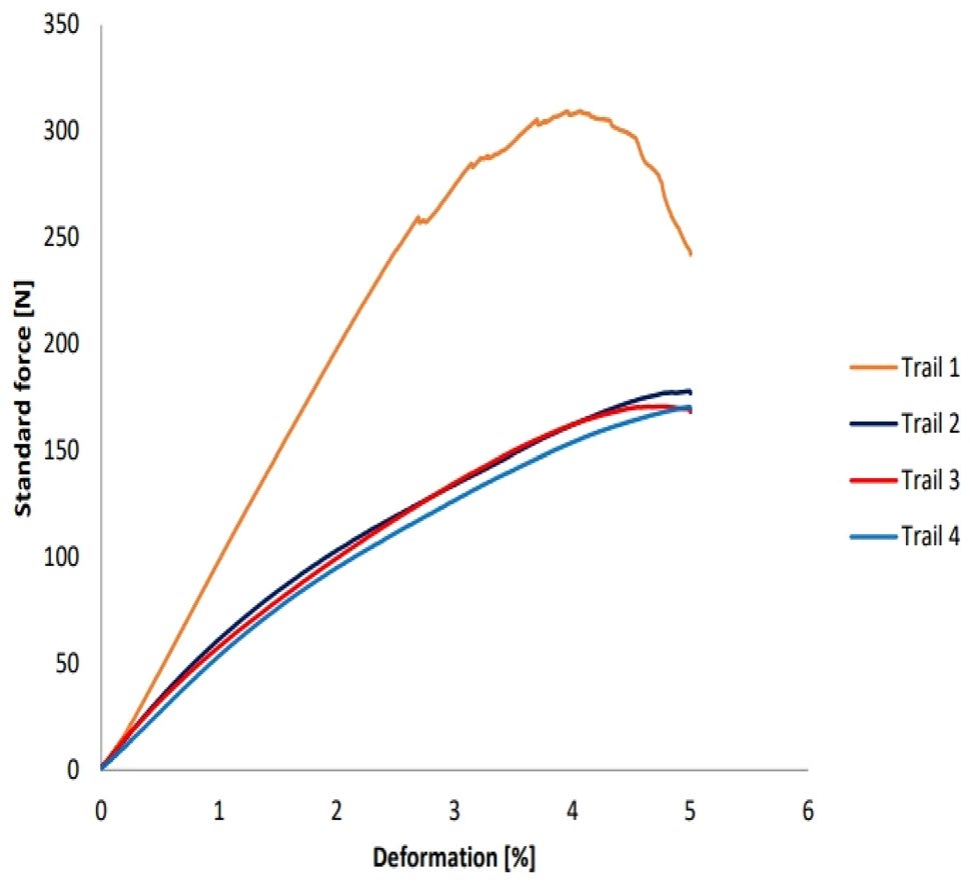
Bending test cycle of MMCS sample.

**Figure 13:**
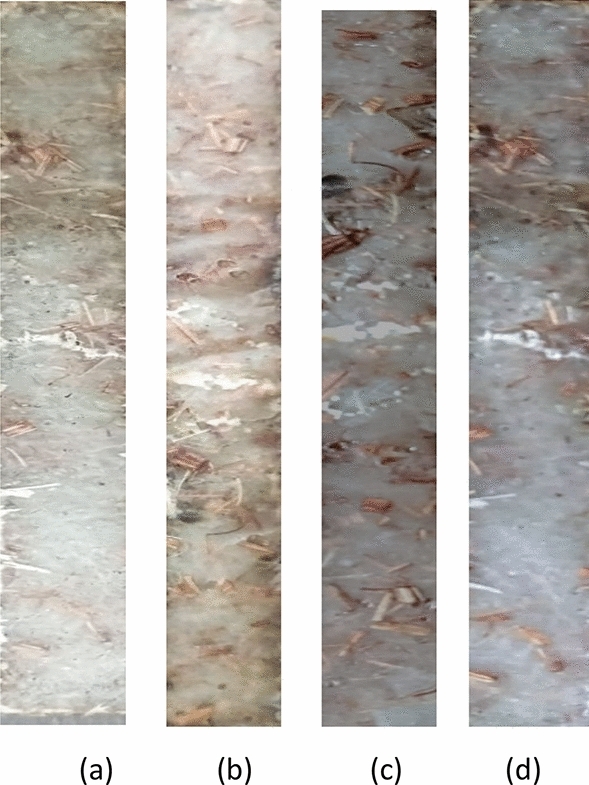
Post bending test sample for all 4 cycles from (**a**) end of first cycle (**b**) end of second cycle (**c**) end of third cycle (**d**) end of fourth cycle.

The sample used for conducting bending test for the first cycle (MMCS) was destructed further through bending load by keeping all the testing parameters kept constant for consecutive 3 cycles to investigate the fracture load and failure mode of the sample. The interesting fact that experiment reveals that is modified matrix sample stands out with unique way of non-breakable trend once it reaches the maximum value. Table 5 depicts the retrieving nature of powdered calamus stem which alters the matrix to behave in a ductile passion and found to be un breakable. The observed deformation moves till it reaches the datum of the support records the maximum value of load taken by the sample but the moment sample is released from the loading setup, sample is retrieved back to its normal geometry. Maximum load taken by the same set of samples for each trial is represented in Fig. 12. Post bending test samples for each cycle witnessing no change in geometry is as shown in Fig. 13. This phenomenon of retrieving from the bending geometry to normal shape is found to be rare and no existing natural fibres depicts the pure ductile nature of the MMCS. Failure analysis of the said case is discussed below with the support of SEM images. Load taking capacity and bending Strength was reduced by 42.5% & 41.64% at the second cycle. Continuing for third cycle, similar parameters were reduced by smaller values reporting that 4.29% and 4.18% respectively.

**Table 5:** Details of the cyclic bending test conducted on MMCs.

Sampling index	Number of trail cycles	Max load (N)	Flexure modulus (MPa)	Ultimate flexure strength (MPa)
Modified matrix laminate (MML)	First	309.4 ± 6.33	3490.1 ± 39	128.2 ± 2.45
	Second	177.9 ± 2.51	2890 ± 57	74.9 ± 1.51
	Third	170.6 ± 2.61	2810 ± 58	71.8 ± 1.54
	Fourth	170.4 ± 2.91	2240 ± 43	71.8 ± 1.54

For the fourth cycle, load taking ability and bending strength reports no further reduction in values observed for the same sample tested consecutively for 4 cycles ensuring the material performing through retrieving it’s the strength and witness no fracture on the loading point. This mode of gaining original shape soon after reaching the maximum deformation for every cycle and no reduction in values at the end of 3^rd^ cycle would be specific in discussion while dealing with the performance of natural fibre-based composites. Additionally, this phenomenon was never been observed for till reported research in composite materials streams witnessing the present work stands with unique way of resembling retrieving strength of novel calamus species of natural fibre composites.

#### Analysis using SEM

**Figure 14:**
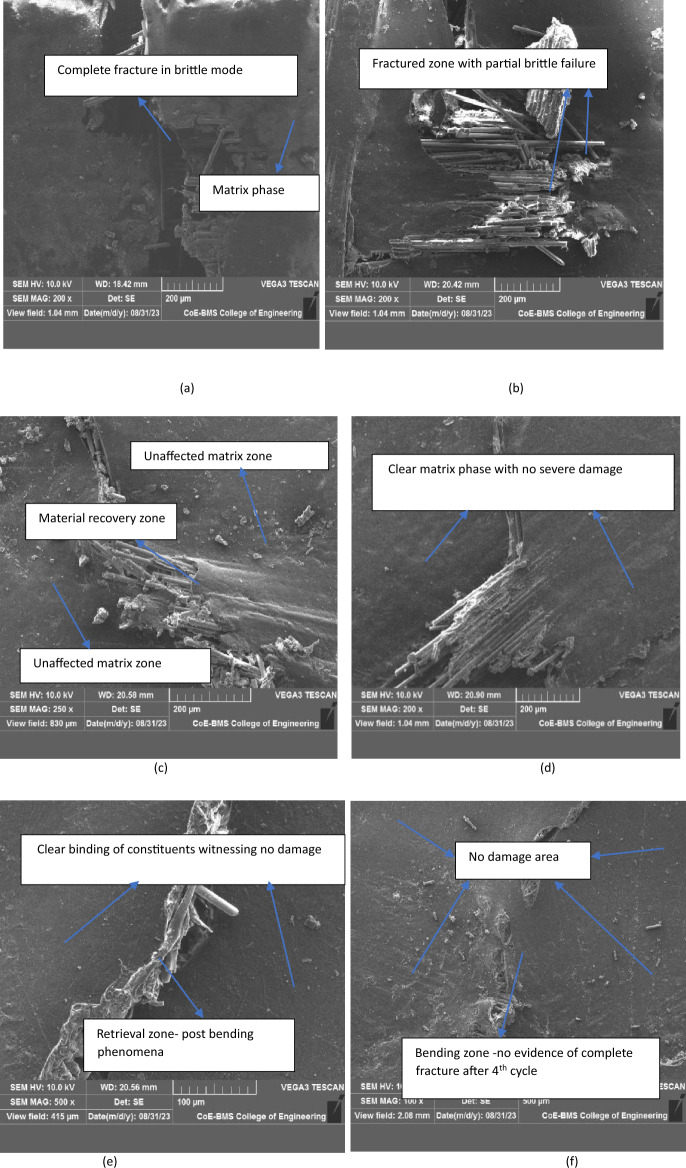
Fractured surface of (**a**) NCS & (**b**) MRCS. For MMCS (**c**) end of first cycle (**d**) end of second cycle (**e**) end of third cycle (**f**) end of fourth cycle.

Above microstructure resembles the fractured surfaces of composite samples tested for bending load. complete fracture in brittle mode is observed for NCS is shown in Fig. 14a highlighting that there is no room for restricting the deformation of the sample once it takes maximum bending load. Similar trend is observed in Fig. 14b where the complete breaking of the sample under bending load is observed for MRCS attaining the least value of bending force. Fibre debris was observed in the vicinity of fractured path. As the glass fibre tends to attain the brittle mode of fracture makes the calamus fibres to split and damage at the point of loading and direction of deformation. Better value of bending strength and modulus for MMCS is due to the fact that uniformity was achieved in filler distribution with matrix and also its observed that no evidence of voids is observed in the microstructure with minimal localized fibre pull out.

Figure 14c highlighting the retrieving nature of powdered calamus stem which alters the matrix to behave in a ductile passion and found to be un breakable. Continued to first bending cycle for the same tested sample, second cycle of bending test was done highlighting the new set of results with 40% reduction in load taking capability compare to the first but the trend of retrieving its geometry was remain unchanged shown in Fig. 14d and the similar trend for further cycle for third and fourth cycle depicts the same behaviour of material where the micro structure of the same is as shown in Fig. 14e,f.It’s found from the SEM analysis is that damage area for each trial tested is tend to be localized leads to no damage or surface rupture was noticed which found to be interesting and surface tends to maintain clear and neat surface once testing done.

### Impact testing

**Figure 15:**
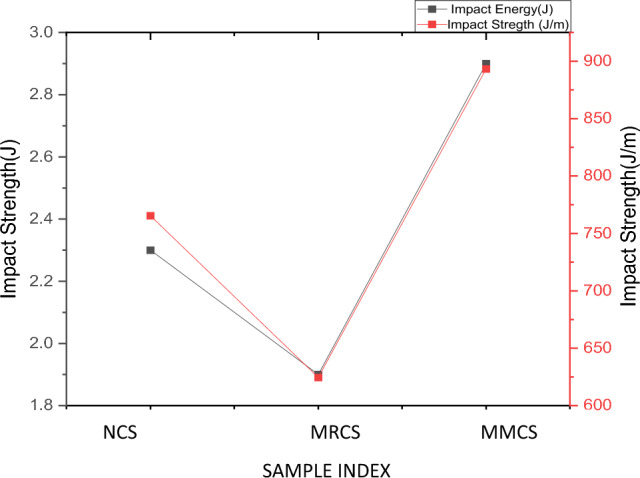
Comparison of Impact Characteristics.

To measure the impact strength of the developed samples, Izod impact test has been carried out using pendulum impact device. Comparison of the results was made with plotted impact characteristics as shown in Fig. 15. Recorded test results are tabulated in the Table 6. Obtained experimental results are convincing over the pure samples where the modified matrix laminates (MMCS) samples depict higher energy absorption value out of other two combinations. It is observed that MML samples reports 26.08% and 52.65% higher than pure samples and modified reinforcement laminates (MRCS) samples respectively. This is due to the fact the composite samples with greater value of strength and modulus yield lower impact values which deforms at lower values which also found in consistent with the results obtained for the study^15^. Performance of MMCS against impact loading is also due to the fact that they exhibit better interfacial bonding between the fibers and matrix and can resist the impact load significantly over other two combinations, similar hypothesis was reported for the work presented previously^18,22^. It is observed that this composition yields nearly 16.71% and 43% higher value of impact strength than pure and MRCS samples respectively. Present results witness the higher value of impact strength as compare to the previous work based on coir fibre-based composites with varied fiber percentage on thermosets^22,23^.

**Table 6:** Results obtained from the impact test.

Sample index	Absorbed energy (J)	Impact strength (J/m)
Neat sample (NCS)	2.3 ± 0.04	765.4 ± 14.3
Modified Reinforcement (MRCS)	1.9 ± 0.01	624.5 ± 12.31
Modified Matrix laminates (MMCS)	2.9 ± 0.05	893.3 ± 17.02

#### Impact test characterization

**Figure 16:**
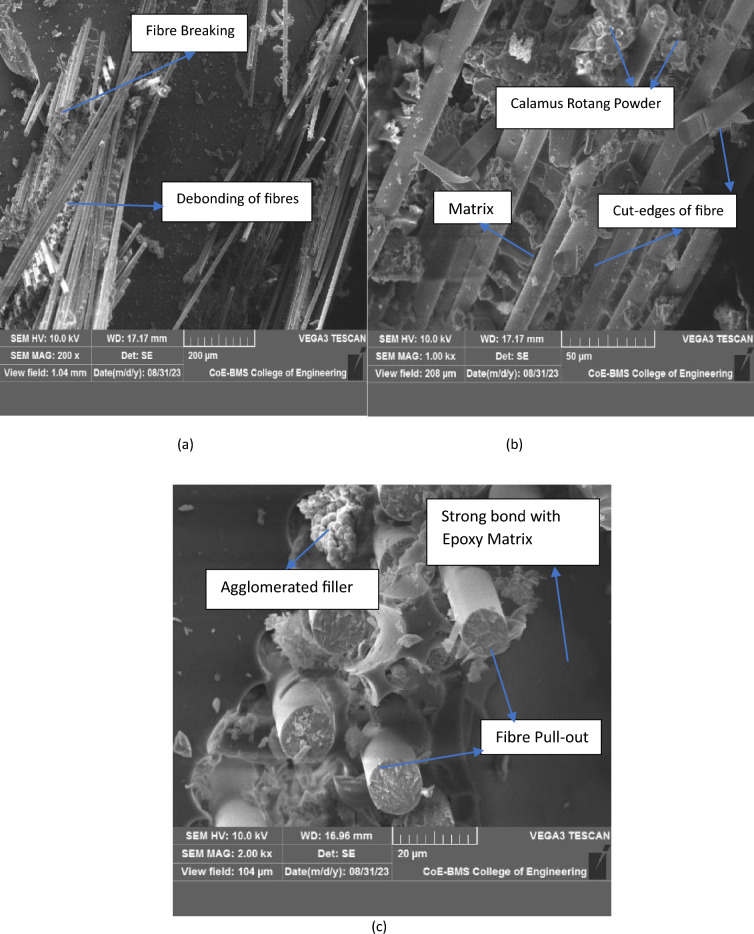
Fractured surface of (**a**) MRCS and (**b**, **c**) MMCS.

Microstructure of samples tested for impact loading is depicted below. Fractured sample due to impact loading for MRCS as shown in Fig. 16a. MRCS records least value of impact force and attains severe damage across the surface is observed. Fibre breaking and splitting of fibres with detachment from matrix system is observed. Failure mode of MMCS is as shown in Fig. 16b where in tip of fibres with sharp cut edges was observed without allowing the matrix to debond with reinforcement instantly which further witness the role of *C. rotang* powder on matrix bonding with reinforcement. Present analysis also found in consistent with the results obtained reporting the better fibre matrix interfacial interaction due to excellent interfacial adhesion between the fibre and the polymers^15^. Bundled reinforcement with fiber pulls out was clearly noticed in the Fig. 16c.

### Interlaminar shear strength testing (ILSS)

**Figure 17:**
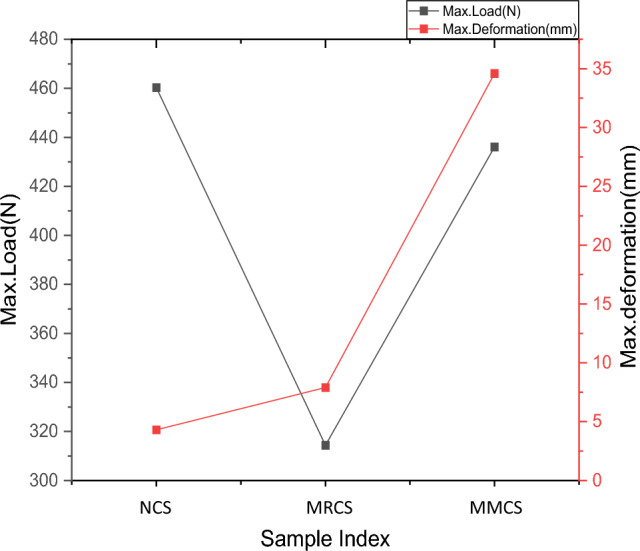
Comparison of ILSS characteristics.

Table 7 depicts the obtained ILSS test results for the 3 sets of samples and the comparison of ILSS parameters of powder, pure and thread composition is reflected from the Fig. 17. Interlaminar shear strength test has been conducted to evaluate the binding ability of the constituent elements, significant observations are made in 3 sets of samples where powder composition proves the maximum load taking against the binding test operation is 38.7% better as compare to threaded sample, however the pure sample shows the maximum value as it had with no modifications done in its compositions as compare to other sets. This maximum ILSS load dictates material strength in the same passion as it delivers the inter laminar chemistry between the fibre, reinforcement and modified elements which proves to be better for powdered sample.

**Table 7:** Results obtained from ILSS test.

Sample index	Max. load (N)	Max. deformation (mm)
Neat sample (CGFSM)	460.28 ± 9.14	4.3 ± 0.08
Modified Reinforcement (MRL)	314.34 ± 6.33	7.9 ± 0.14
Modified Matrix laminates (MML)	436.08 ± 8.03	34.59 ± 7.2

#### ILSS characterization

**Figure 18:**
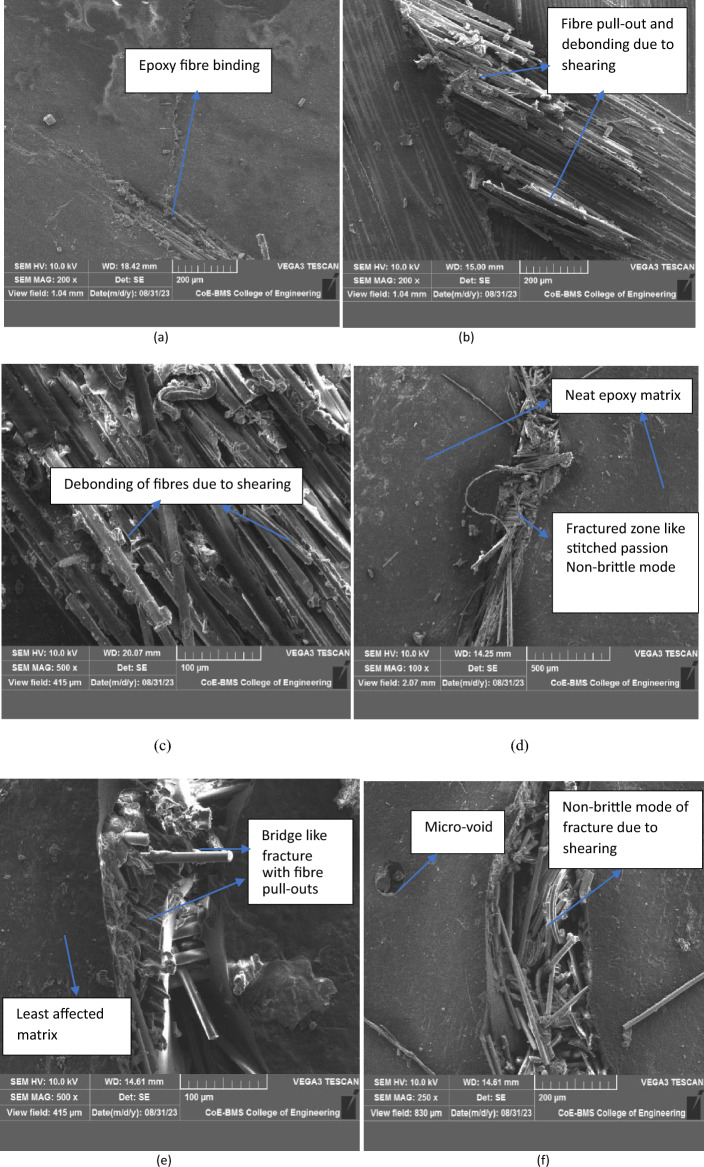
ILSS tested samples (**a**) NCS, (**b**, **c**) MRCS, (**d**–**f**) MMCS.

Interlaminar Shear Strength was investigated for prepared CS’s. Below microstructure resembles the failure mode of tested samples. Neat composite sample depicts the clear bonding and perfect bonding of glass fibres with epoxy system shown in Fig. 18a whereas debonding and surface damage through shearing of the laminates and cohesion between hybrid fibres and matrix were clearly noticed in MRCS which is as shown in Fig. 18b,c.Resistance to interlaminar shearing was unlikely to MRCS for the combination MMCS shown in Fig. 18d,e where resistance to interlaminar breaking was noticeable as modified matrix combination builds better interlayer adhesion against shearing . The breakage of fibres with the binding ground was not instant and sufficiently notice the ductile nature of *C. rotang* powder which alters the failure of reinforcement like bent fibres which is as shown in Fig. 18f.

## Conclusion

Present study investigates the effect of novel species of *C. rotang* stem fibers as potential reinforcing element in order to the evaluate the suitability to fit in to global composite industry for various structural applications in aircraft, automobile etc. Based on experimental observations, conclusions are drawn with respect to three different sets of samples. It’s concluded that, for tensile test, the maximum load taken by pure sample is 64.8% and for powder sample is 36.4% greater than the load taken by thread sample. Obtained tensile modulus and tensile strength for pure and powder is 24.1%, 51.3% and 86%, 48.3% higher as compare to the thread samples respectively. In flexure test, the maximum load taken by pure sample is 21.7% and for powder sample is 35.2% greater than the load taken by thread sample, hence obtained flexure modulus and flexure strength for pure and powder is 37.9%, 33.7% and 73.4%, 13.2% higher than the threaded sample respectively. Also, for obtained impact strength, it is observed that powdered sample yield nearly 16.71% and 43% higher value of impact strength as compare to pure and thread samples respectively. From ILSS test, maximum shear load for pure and powder sample is 46.4% and 38.7% respectively greater than the thread sample. The significance of the present work concluded with novel findings as retrieval strength of *C. rotang* based natural fibre composite (MMCS). Though the load bearing ability and strength reduced by 42.5% & 41.64% at the end of the second cycle, the trend of declining strength was reduced noticeably for the third cycle and remain constant at the end of the fourth cycle witnessing the material retrieving strength and also reflects no fracture on the loading point.

## Data Availability

The datasets used and/or analysed during the current study available from the corresponding author on reasonable request.
